# Interactions Between Sarcopenia, Physical Frailty and Resting Energy Expenditure in Cirrhosis and Portal Hypertension †

**DOI:** 10.3390/nu17243844

**Published:** 2025-12-09

**Authors:** Rachael Jacob, Joanne Craik, Aviv Pudipeddi, Laura Park, Grace Aw, Natalie L. Y. Ngu, Prahalath Sundaram, Helen Vidot, Talal Valliani, Madeleine Gill, Dominic Staudenmann, David Bowen, Simone I. Strasser, Geoffrey W. McCaughan, Ken Liu

**Affiliations:** 1 AW Morrow Gastroenterology and Liver Centre, Royal Prince Alfred Hospital, Sydney 2050, Australia; rachael.jacob@health.nsw.gov.au (R.J.); prahalath.sundaram@health.nsw.gov.au (P.S.); talal.valliani@health.nsw.gov.au (T.V.); maddie.gill@gmail.com (M.G.); david.bowen1@health.nsw.gov.au (D.B.); simone.strasser@health.nsw.gov.au (S.I.S.); g.mccaughan@centenary.org.au (G.W.M.); 2 Concord Repatriation General Hospital, Sydney 2139, Australia; aviv.pudipeddi@health.nsw.gov.au; 3 Faculty of Medicine and Health, University of Sydney, Sydney 2050, Australia; 4 Department of Nutrition and Dietetics, Royal Prince Alfred Hospital, Sydney 2050, Australia; craikjk@outlook.com (J.C.); helen.vidot@health.nsw.gov.au (H.V.); 5 Alfred Health, Melbourne 3004, Australia; laura.sv.park@gmail.com; 6 Department of Medical Imaging, Royal Brisbane and Women’s Hospital, Brisbane 4006, Australia; grace.aw@health.qld.gov.au; 7 Monash Health, Monash University, Melbourne 3800, Australia; natalielyngu@gmail.com; 8 Flinders Medical Centre, Adelaide 5042, Australia; 9 Intesto-Gastroenterologische Praxis & Crohn-Colitis Zentrum, 3012 Bern, Switzerland; staudenmannd@intesto.ch; 10 Liver Immunology Program, Centenary Institute, Sydney 2050, Australia; 11 Liver Injury and Cancer Program, Centenary Institute, Sydney 2050, Australia

**Keywords:** sarcopenia, frailty, indirect calorimetry, cirrhosis, portal hypertension, liver transplantation

## Abstract

**Background/Objectives:** Sarcopenia and frailty are prevalent and independently prognostic in cirrhosis. Few studies have evaluated both together to ascertain their interaction and phenotypic differences. None have studied their relationship with resting energy expenditure (REE). We simultaneously examined sarcopenia, frailty and REE in a cohort of patients with cirrhosis and portal hypertension—a novel approach. **Methods:** We retrospectively studied consecutive patients with cirrhosis and portal hypertension, prospectively recruited between 2015 and 2018 to undergo sarcopenia (transversal psoas muscle thickness [TPMT]/height), frailty (Fried Frailty Index [FFI]), and REE assessments via indirect calorimetry. The primary outcome was transplant-free survival (TFS). **Results:** Ninety-seven patients were recruited with sarcopenia and frailty present in 26% and 40%, respectively. Patients with sarcopenia or frailty alone were phenotypically similar except those with sarcopenia had lower median body mass index (BMI) (23 vs. 28 kg/m^2^, *p* = 0.032) and were more likely to be hypermetabolic (60% vs. 0%, *p* = 0.017). Median TFS was lower in patients with sarcopenia (3.6 months) or frailty (4.5 months), compared to those with neither (10.3 months), while patients with both sarcopenia and frailty exhibited the worst TFS (1.8 months, log-rank *p* = 0.001). Independent predictors of death or liver transplant were sarcopenia, hepatic encephalopathy, and a higher model for end-stage liver disease score. **Conclusions:** In patients with cirrhosis and portal hypertension, sarcopenia and physical frailty are related but have differences in BMI and REE. The deleterious impact of sarcopenia and frailty on TFS are additive. Sarcopenia remains an independent predictor of TFS after adjusting for frailty.

## 1. Introduction

Sarcopenia and frailty are common in advanced liver disease, occurring in 40–70% [1,2,3] and 17–43% [4,5,6,7,8,9], respectively. Sarcopenia refers to a progressive and generalised depletion in skeletal muscle mass, strength, and function [10]. Global frailty represents a complex syndrome defined as a state of decreased physiological reserve with increased vulnerability to health stressors [10]. It is a multidimensional term encompassing physical, psychosocial and social domains, with its origins in geriatrics literature. However, the majority of hepatology literature on frailty, including our present study, focuses on the *physical* component of global frailty [10]. This has been operationalised as impaired muscle contractile function. Sarcopenia and physical frailty are distinct but related entities, although they are often referred to interchangeably in clinical practice. While sarcopenia is a major contributor to and often co-exists with frailty, the two can occur independently. Importantly, sarcopenia and frailty have been shown to be robust predictors of adverse clinical outcomes in cirrhosis including hepatic decompensation, development of acute-on-chronic liver failure (ACLF), increased and prolonged hospitalisations, reduced quality of life, and increased mortality [5,6,11,12,13,14].

Malnutrition is also prevalent in advanced liver disease, affecting 20–50% of patients [15]. As malnutrition accelerates skeletal muscle loss, accurate macronutrient supplementation tailored to individual energy requirements is essential [16]. In cirrhosis patients, indirect calorimetry is the gold standard for estimating energy expenditure and is superior to predictive equations commonly utilised in clinical practice [17,18]. Indirect calorimetry provides a reliable estimate of resting energy expenditure (REE), which constitutes 50–70% of total energy expenditure, enabling precise assessment of a patient’s energy requirements [18].

While most studies have examined the impact of either sarcopenia *or* frailty on outcomes in cirrhosis, surprisingly, very few have looked at both together. Hence, it remains unclear whether any discernible phenotypic differences exist between patients with sarcopenia and those with frailty. For instance, it is not known if a patient who is sarcopenic but not frail has similar or different characteristics to a patient who is frail but not sarcopenic. Likewise, it is not known whether the poorer prognosis associated with sarcopenia and frailty are additive when they coexist in the same patient. Without studying both entities together, it is not possible to ascertain if they are predictors of clinical outcomes independent of each other, or simply collinear variables. Furthermore, to our knowledge, no previous studies have performed sarcopenia, frailty, and indirect calorimetry simultaneously on the same population. Since both sarcopenia and frailty are potentially modifiable with nutritional intervention [19], determining the REE and fuel utilisation of cirrhosis patients with sarcopenia versus frailty versus both is not only novel, but of therapeutic importance to guide the prescription of nutritional supplementation.

Therefore, for the aforementioned reasons, we aimed to characterise the prevalence, characteristics and impact on clinical outcomes of sarcopenia, frailty and REE in a cohort of patients with cirrhosis and portal hypertension.

## 2. Materials and Methods

### 2.1. Study Population

This retrospective analysis included outpatients attending liver clinics at Royal Prince Alfred Hospital, a state-wide liver transplant (LT) referral centre, prospectively recruited to undergo sarcopenia, frailty and nutritional assessments between April 2015 and August 2018. Adult (age ≥ 18 years) patients with a confirmed diagnosis of cirrhosis and portal hypertension were included. Consecutive patients were approached to reduce selection bias. Both LT-eligible and -ineligible patients were recruited. Cirrhosis was defined by liver histology, radiologic evidence (i.e., coarse liver echotexture with nodularity and small liver size), and/or alanine aminotransferase-based liver stiffness measurement [20] or transient elastography [21]. Portal hypertension was defined by the presence of clinical (i.e., ascites, splenomegaly, caput medusae), radiologic (i.e., ascites, abdominal varices, splenomegaly, recanalised umbilical vein), or endoscopic features (i.e., gastro-oesophageal varices). Patients were excluded if they had prior LT or could not complete frailty assessment and/or indirect calorimetry due to the inability to follow instructions. Patients with concomitant hepatocellular carcinoma (HCC) were not excluded. Patient demographic, clinical, radiologic and laboratory data were obtained from electronic medical records. The study was conducted according to the Declaration of Helsinki and was approved by the Sydney Local Health District Human Research Ethics Committee (2020/ETH00798). Informed consent was obtained from all participants.

### 2.2. Sarcopenia, Frailty and Nutritional Assessment

Sarcopenia was assessed radiologically by measuring the right transversal psoas muscle thickness (TPMT) at the level of the umbilicus on computed tomography (CT) scans and normalising it to the patient’s height as previously described [22]. A TPMT/height cut-off of ≤16.8 mm/m was used to define sarcopenia [22]. CT scans were only performed as part of routine care (i.e., for HCC surveillance/diagnosis, LT work-up, or if otherwise clinically indicated) and no additional scans were performed as part of this study. Thus, sarcopenia measurements were not available for the entire study cohort.

The baseline date was defined as the date when frailty assessment was performed. Because the commencement of this study predated development of the Liver Frailty Index (LFI), frailty was assessed using the Fried Frailty Index (FFI) as previously described [23]. The FFI has been validated in cirrhosis and measures physical frailty. It comprises five self-reported and performance-based measures assessing slowness, exhaustion, physical activity, unintentional weight loss, and weakness. Patients were classified as frail if they scored ≥ 3/5 on the FFI. For analysis, patients were categorised into four groups based on their sarcopenia and frailty status: not frail and not sarcopenic (NFNS), not frail but sarcopenic (NFS), frail but not sarcopenic (FNS), and frail and sarcopenic (FS).

Comprehensive nutritional assessment included anthropometric measurements, malnutrition screening, and performance of indirect calorimetry using portable devices. Anthropometric measurements included weight, body mass index (BMI), mid-arm circumference (MAC), mid-arm muscle circumference (MAMC), triceps skinfold thickness (TSF), and hand grip strength (HGS). Malnutrition screening was performed using a validated Subjective Global Assessment (SGA) questionnaire [24].

### 2.3. Indirect Calorimetry

All patients were offered indirect calorimetry using a COSMED Quark machine (COSMED, Rome, Italy) to assess REE and fuel utilisation. Patients who agreed presented in the morning following a 10–12 h fasting period and the metabolic cart was set up in a quiet, private room with soft lighting. As per protocol, the first ten minutes of readings were discarded to allow patients to recover from the activity of arriving at the clinic and to rest in bed. Patients were prevented from sleeping during the assessment. Monitoring continued until a steady-state measurement was obtained, identified by a five-minute period were the co-efficient variations for both vCO_2_ and vO_2_ were less than 10%. Each patient was asked to complete a three-day food diary to assess their current oral intake, which was analysed by the dietitian (JC) using FoodWorks software (Version 9, Xyris Pty Ltd., Brisbane, Australia) and compared to the calorimetry results for energy expenditure (EE) and fuel utilisation. Patients were classified as hypermetabolic, normometabolic, or hypometabolic based on comparison of measured REE using indirect calorimetry to predicted EE using the Harris-Benedict Equation [25]. Hypermetabolism was defined as REE exceeding the predicted value by ≥20%, normometabolism as REE within 20% of predicted value and hypometabolism as REE below the predicted value by >20% [25]. An average urinary nitrogen of 12.3 g N/day was used to calculate fuel utilisation (average is based on 24 h urinary nitrogen excretion studies using Kjeldahl analysis in individuals with cirrhosis from our unit).

This article is a revised and expanded version of a paper entitled ‘Interactions between sarcopenia, frailty and resting energy expenditure in patients with cirrhosis and portal hypertension’, which was presented at Australian Gastroenterology Week, Adelaide, Australia, September 2024 [26].

### 2.4. Statistical Analysis

Continuous variables were expressed in means with standard deviation (SD) or medians with interquartile range (IQR) as appropriate. Differences between subgroups were analysed using χ^2^ or Fisher exact test for categorical variables and Student’s *t*-test or Mann–Whitney U test for continuous variables as appropriate. Correlations between sarcopenia and frailty and different variables were determined by Spearman’s correlation coefficient analysis. The primary outcome of interest was transplant-free survival (TFS, i.e., time to death or LT). TFS was estimated using the Kaplan–Meier method with log-rank test to determine statistical significance between study groups. Univariable analyses were conducted on collected variables to determine clinical parameters associated with death or LT. Multicollinearity between covariates was assessed with a variance inflation factor (VIF); values exceeding five were deemed significant. A multivariable Cox regression model using a backward stepwise approach was performed among covariates that were associated with death or LT on univariable analysis (*p* < 0.1). Hazard ratio and 95% confidence interval (CI) of the risk factors were calculated. All statistical analyses were performed using IBM Statistical Package for Social Science (SPSS version 27.0, Armonk, NY, USA). Statistical significance was taken as *p* < 0.05.

## 3. Results

### 3.1. Patient Characteristics

During the study period, 97 patients underwent a combination of sarcopenia, frailty, and nutritional assessments. A total of 96 (99%) completed frailty assessments, 90 (93%) had CT sarcopenia measurements and 63 (65%) patients had indirect calorimetry performed. Reasons for incomplete assessments are outlined in Supplementary Table S1. Over half the cohort (59/97, 61%) had all three measurements (sarcopenia, frailty and indirect calorimetry) performed. The median times between frailty and sarcopenia measurements, frailty and REE measurements, and sarcopenia and REE measurements were 1.5 (IQR 0.2–4.1), 1.4 (IQR 0.2–3) and 2.8 (IQR 0.9–5) months, respectively. Baseline characteristics of patients are presented in Table 1. Patients were predominantly male (71%) with a median age of 57 (IQR 52–63) years and median Child–Pugh and model for end-stage liver disease (MELD) scores of B9 (IQR 7–10) and 16 (IQR 11–20), respectively. The most common aetiologies of liver disease were chronic hepatitis C (37%), alcohol use disorder (23%), autoimmune liver disease (i.e., autoimmune hepatitis, primary biliary cholangitis and primary sclerosing cholangitis) (21%) and metabolic dysfunction-associated fatty liver disease (10%). Other aetiologies included chronic hepatitis B (5%) and other less frequent causes including cryptogenic liver disease, secondary biliary cirrhosis, and erythropoietic protoporhypria) (3%). Nineteen (20%) patients had concomitant HCC. The majority (67%) of patients had ascites and 34% had experienced a previous episode of hepatic encephalopathy.

### 3.2. Study Measurements

According to the FFI, 40% (38/96) of patients were frail while 26% (23/90) were sarcopenic according to TPMT/height measurements. Median FFI score was 2 (IQR 1–3) and median TMPT/height was 20 mm/m (IQR 17–22). Of the 89 patients with both sarcopenia and frailty assessments, 52% were NFNS, 23% FNS, 16% FS, and 9% NFS.

**Figure 1:**
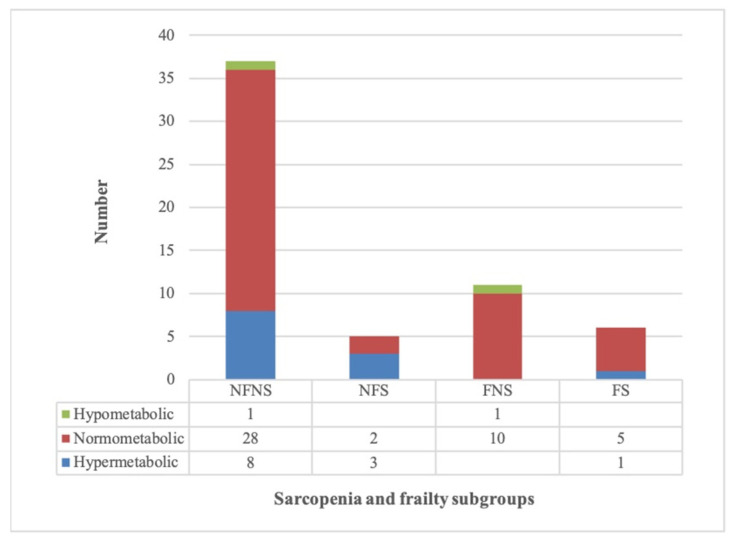
Resting energy expenditure in patients with complete sarcopenia, frailty, and calorimetry assessments (*n* = 59). **Abbreviations**: NFNS, not frail and not sarcopenic; NFS, not frail but sarcopenic; FNS, frail but not sarcopenic; FS, frail and sarcopenic.

The median predicted and measured REE were 1637 (IQR 1440–1799) and 1695 (IQR 1459–1955) kcal/day, respectively. Accordingly, the majority of patients were normometabolic (48/63, 76%), thirteen (21%) were hypermetabolic and two (3%) were hypometabolic. Among the 59 patients with complete frailty and sarcopenia assessments, a similar distribution was observed across subgroups (Figure 1).

The median respiratory quotient (RQ) was 0.8 (IQR 0.7–0.9) and the median percentage of carbohydrate, fat and protein fuel utilisation were 23 (IQR 8–40), 55 (IQR 39–69) and 20 (IQR 17–23) respectively. These results demonstrate fat utilisation as the main fuel source, along with increased protein and decreased carbohydrate utilisation, as expected in cirrhosis patients [27].

Malnutrition screening [24] was performed in 77% (75/97) of patients. In terms of nutritional status, 23% of patients were well nourished (SGA A), 55% were mild–moderately malnourished (SGA B) and 23% were severely malnourished (SGA C). Median MAC, MAMC and TSF were 285 (IQR 266–330), 249 (IQR 224–271) and 12 (IQR 8–19) mm respectively. Median HGS was 28 kg (IQR 21–35).

### 3.3. Interactions Between Sarcopenia, Frailty, and Energy Expenditure

There was a modest but significant inverse correlation between TPMT/height and FFI (Spearman *rho* = −0.266, *p* = 0.016) (Table 2 and Table 3). Thus, lower patient muscle mass is associated with higher frailty score. FFI (*rho* = −0.308, *p* = 0.008) correlated inversely with REE; that is, a higher frailty score is associated with reduced REE. No significant correlation was observed with TPMT/height (*rho* = 0.224, *p* = 0.088). Both FFI (*rho* = 0.333, *p* = 0.008) and TPMT/height (*rho* = −0.310, *p* = 0.017) correlated with increased protein fuel utilisation but not carbohydrate or fat utilisation. This suggests that higher frailty measurements and lower muscle mass are associated with increased protein fuel utilisation.

To establish if sarcopenia and frailty present with different phenotypes, we compared baseline characteristics of patients who were sarcopenic but not frail (NFS group, *n* = 8) with those who were frail but not sarcopenic (FNS group, *n* = 21). Compared with FNS, NFS patients had a significantly lower median BMI (23 vs. 28 kg/m^2^, *p* = 0.032). NFS patients were also more likely to be hypermetabolic (60% vs. 0%, *p* = 0.017) (Table 4). Though there was a greater proportion of FNS patients who were normometabolic compared to NFS patients, this did not reach statistical significance (91% vs. 40%, *p* = 0.063). Characteristics were otherwise similar between the two groups with regards to demographics, liver function, anthropometric measurements, and REE (Table 4).

### 3.4. Other Associations with Sarcopenia and Frailty

TPMT/height correlated directly with BMI, sodium, MAC, MAMC, and HGS and inversely with Child–Pugh score, MELD score and SGA (Table 2). Thus, sarcopenia (as suggested by lower TPMT/height) is significantly associated with reduced BMI, HGS, anthropometry measurements, worse liver function (as measured by Child–Pugh and MELD scores), hyponatraemia and malnutrition (as judged by SGA).

FFI correlated directly with international normalised ratio (INR), Child–Pugh score, MELD score, and SGA and inversely with sodium, MAC, MAMC, and HGS (Table 3). Thus, frailty (as suggested by higher FFI) is significantly associated with worse liver function (as measured by INR, Child–Pugh and MELD scores), hyponatraemia, malnutrition (as judged by SGA) and reduced BMI, HGS, and anthropometry measurements.

### 3.5. Transplant-Free Survival

**Figure 2:**
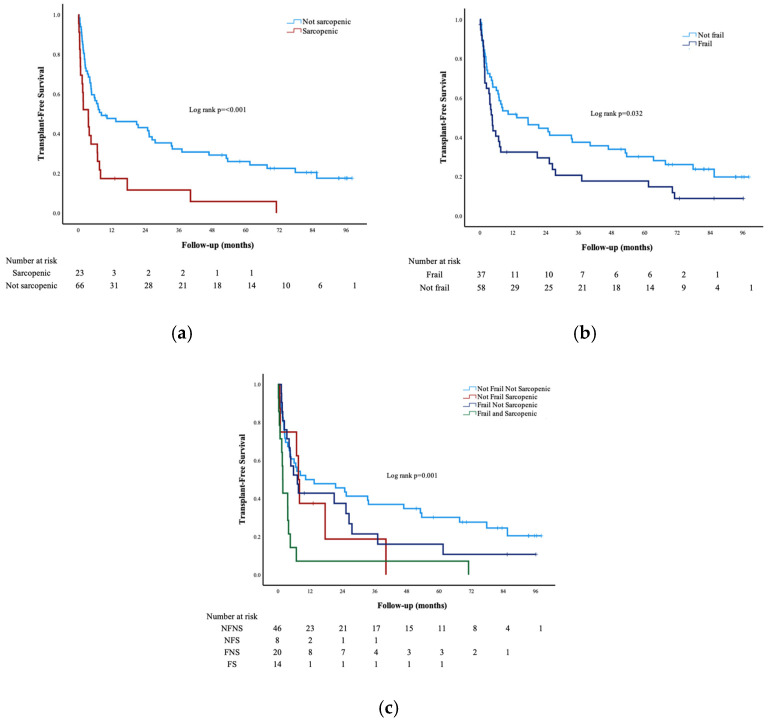
Kaplan–Meier survival curves stratified by (**a**) sarcopenia status, (**b**) frailty status, and (**c**) frailty and/or sarcopenia status. **Abbreviations**: NFNS, not frail and not sarcopenic; NFS, not frail but sarcopenic; FNS, frail but not sarcopenic; FS, frail and sarcopenic.

After a median follow up of 5.75 years (IQR 2.2–7.6), 50 (51%) patients underwent LT and 35 (36%) patients died. The median overall TFS was 7.2 months (95% CI 4.8–9.6). By Kaplan–Meier analysis, median TFS was significantly lower in patients who were sarcopenic (3.6 vs. 8.3 months; *p* ≤ 0.001) or frail (4.5 vs. 13.4 months; *p* = 0.032) (Figure 2a,b). When comparing all subgroups, sarcopenia and frailty had a cumulative impact, with the lowest median TFS seen in the FS (1.8 months, 95% CI 1.4–2.1) compared to the NFNS, NFS and FNS groups (log-rank *p* = 0.001) (Figure 2c). Since CT scans were not always performed at or close to the time of frailty assessment, we performed a sensitivity analysis excluding patients who did not have CT scans within six months (180 days) of their frailty assessment. This revealed similar results as the original analyses (Supplementary Figure S1). Similarly, another sensitivity analysis excluding patients with HCC also did not change our main findings (Supplementary Figure S2).

Univariable predictors of death or LT were alcohol-associated liver disease, metabolic-dysfunction associated liver disease, presence of ascites, hepatic encephalopathy, increased MELD score, increased FFI score and lower TPMT/height (Table 5). On multivariable analysis, independent predictors of death or LT were the presence of hepatic encephalopathy (adjusted hazard ratio [aHR] = 2.051, 95% CI 1.211–3.474, *p* = 0.008), increased MELD score (aHR = 1.114 per point increase, 95% CI 1.073–1.156, *p* < 0.001) and reduced TPMT/height (aHR = 0.930 per mm/m increase, 95% CI 0.868–0.996, *p* = 0.037).

## 4. Discussion

The global burden of cirrhosis is increasing and patients with decompensated disease and/or portal hypertension have a worse prognosis than those with compensated disease [28,29]. Although Child–Pugh and MELD scores are good prognosticators in cirrhosis, they do not account for sarcopenia or frailty which are both predictors of mortality independent of liver function [6,30].

We present the first study to our knowledge to simultaneously evaluate sarcopenia, frailty, and REE as well as other nutritional assessments in patients with cirrhosis and portal hypertension. Few studies have assessed sarcopenia and frailty together in the same cohort and none have adjusted for both within the same multivariable analysis [31,32]. Thus, their relative contributions to increased mortality are not known. Indeed, international guidelines have recommended the inclusion of both frailty and sarcopenia as complementary endpoints in future research [10]. We observed that sarcopenic and frail patients were phenotypically similar other than a lower median BMI and higher rates of hypermetabolism in the former. The detrimental effects of sarcopenia and frailty were additive with FS patients having the worst TFS compared to NFS, FNS, and NFNS subgroups. Interestingly, on multivariable analysis, frailty was no longer a significant predictor of TFS after adjusting for sarcopenia.

Our Australian cohort presented similarly to those reported elsewhere. First, our event rate of 51% LT and 36% deaths at >5 years was consistent with what would be expected in a cohort of largely decompensated patients [29]. Second, sarcopenia and frailty were found in 26% and 40% of our cohort, respectively. While frailty prevalence in our cohort was similar to other studies (17–43%), sarcopenia was lower than the reported range of 40–70% [1,2,3], likely due to the different radiological definitions of sarcopenia used in slightly different cohorts. We included consecutive patients with cirrhosis and portal hypertension rather than LT waitlist patients. TPMT/height was chosen as it is easily measured without the need for specialised software and has good interobserver agreement between experienced and non-experienced operators [33]. A cut-off of <16.8 mm/m was used to define sarcopenia as it has been shown to be prognostic in patients with refractory ascites and MELD score ≤ 25 which matches our cohort (67% ascites, median MELD score 16) [22]. Furthermore, we found post hoc that TPMT/height was the most prognostic sarcopenia measurement in our cohort when compared to skeletal muscle index (SMI) or total psoas muscle area (TPA), justifying its use in our definition of sarcopenia (Supplementary Tables S2 and S3). Although SMI is often thought to be the gold standard for sarcopenia measurement, curiously, it was not as discriminatory as TPMT/height for predicting TFS in our cohort. One explanation could be because only 26% of patients were identified as sarcopenic by the TPMT/height method vs. 62% identified by the SMI method. These 26% had a median SMI of 38 (IQR 34–41) cm^2^/m^2^ which was lower than the 40 (IQR 36–44) cm^2^/m^2^ in the 62% identified as sarcopenic by SMI. Accordingly, most of these 26% of patients had an SMI in the lowest quartile of the entire cohort. Therefore, TPMT/height was perhaps more discriminatory than SMI in our cohort because it picked out patients with the worst sarcopenia rather than picking out over half the cohort.

As expected, we found sarcopenia and frailty were related with significant correlations between TPMT/height and FFI measurements. Indeed, there were no differences in demographics, liver disease aetiology and severity, and TFS between pure sarcopenic and frail phenotypes. This overlap is not surprising since the study of frailty in cirrhosis has focused on physical frailty and has been operationalised as impaired muscle function, while sarcopenia has been operationalised as impaired muscle mass [34]. It also points to some shared pathophysiological mechanisms including circulation of pro-inflammatory cytokines, hyperammonaemia, and reduced testosterone levels [34]. While it is well-known that sarcopenia and frailty can also exist in isolation, we discovered in this study that NFS patients were significantly more likely to be hypermetabolic (or catabolic) compared to FNS patients, though low numbers in this comparison are acknowledged. Correspondingly, NFS also had significantly lower median BMI than FNS patients and increased frailty scores were associated with reduced REE (hypometabolism). These novel findings highlight that sarcopenic and frail patients have different nutritional requirements. Although nutrition prescription is recommended as part of the management plan in both frailty and sarcopenia guidelines [10], patients with sarcopenia may require a more intensive approach. Thus, the prescription of nutritional support and supplementation needs to be personalised according to each patient’s phenotype and REE [10]. Protein supplementation appears to be particularly important since worsening frailty and sarcopenia were both significantly associated with increased protein utilisation (but not other fuels) in our study. A daily protein intake of 1.2–1.5 g/kg ideal body weight per day has been recommended in guidelines [10].

We observed significantly lower TFS if either sarcopenia or frailty was present, while patients with both frailty and sarcopenia had the worst TFS, indicating an additive prognostic effect. Thus, measuring both frailty and sarcopenia in cirrhosis patients adds another dimension to prognostication compared to assessing either alone. This can help identify patients with the poorest predicted prognosis, in order to prioritise them for clinical review, closer monitoring, (p)rehabilitation programmes, and potentially expedite LT if their frailty and sarcopenia permit or are reversible [9]. On multivariable analysis, only sarcopenia (TPMT/height), the presence of hepatic encephalopathy and MELD score were independent predictors of LT or death after adjusting for other variables including FFI. This suggests that sarcopenia may be a more influential risk factor than frailty, although further studies are required to validate this. In contrast to our findings, a single-centre study of 105 LT waitlist patients by D’Arcangelo et al. demonstrated that frailty (measured by LFI) was independently associated with increased waitlist mortality while sarcopenia (measured by SMI) was not a significant predictor on univariable or multivariable analysis [35]. In another single-centre study of 132 LT waitlist patients, Perdiguero et al. observed that SMI and LFI were both independent predictors of developing ACLF on the waitlist [36]. These discrepancies in the prognostic values of sarcopenia and frailty between the three studies could be explained by differences in study cohorts (LT waitlist patients vs. patients with cirrhosis and portal hypertension) and methods of measuring sarcopenia and frailty (SMI vs. TPMT/height and LFI vs. FFI, respectively). Clearly, larger prospective multicentre studies in both LT and non-transplant settings are required to clarify these mixed data.

The above points highlight the potential usefulness of objectively measuring frailty, sarcopenia, REE and fuel utilisation together in cirrhosis patients as part of standard-of-care (resource permitting) to obtain the full picture of their physical and nutritional status. Ideally, these tests should also be incorporated into the evaluation and prioritisation of LT recipients, in addition to other decision-making tools such as Child–Pugh or MELD scores. Prior to this study, guidelines have recommended that all patients with cirrhosis should be assessed for frailty, whereas the assessment of sarcopenia was to be “considered” and thought to be “useful in select groups” [10]. In 2018, a survey of 41 LT clinicians performed by the American Society of Transplantation reported that whilst 95.6% agreed on the utility of frailty assessment in LT evaluation, only 23.3% performed it routinely [37]. Thus, screening for frailty and/or sarcopenia are currently underperformed in cirrhosis patients and more work is required to raise awareness among clinicians of their importance. Limitations on time and resources in liver clinics also present obstacles [10]; however, the LFI and TPMT/height are relatively quick and easy to perform.

Our study’s strength includes its novelty in being the first to study sarcopenia, frailty and REE simultaneously. Patients were also prospectively recruited and typical of those seen in advanced hepatology clinics. Nonetheless, several limitations deserve mention. First, although patients were prospectively recruited, the analysis was performed retrospectively, subjecting it to bias. Additionally, given the retrospective analysis, there was a lack of data on comorbidities which could potentially influence sarcopenia and/or frailty. Second, our relatively small sample size may have limited the ability to detect significant differences between subgroups and our single-centre cohort may restrict the generalisability of our results. Therefore, although our study has yielded some interesting results, validation is needed in future studies with larger, prospective, multicentre cohorts. Third, not all patients had sarcopenia and indirect calorimetry measurements and measurements were not performed synchronously. We relied on obtaining sarcopenia measurements from CT scans otherwise required for the patients’ standard-of-care to avoid unnecessary ionising radiation exposure. Despite this, the majority of patients were able to have sarcopenia measurements performed (93%) and a sensitivity analysis of those with sarcopenia measurements within six months of their frailty measurement demonstrated similar overall results. Although all patients were invited to undergo indirect calorimetry, the lower rate of uptake (65%) is a reflection of our relatively unwell cohort (median TFS 7.2 months). However, we still demonstrated differences in metabolism between study subgroups. Finally, since the conception of this study, the LFI was developed in 2018, which is a simpler tool than the FFI [38]. While the FFI incorporates both self-reported and performance-based measures, the LFI uses only performance-based assessments. This offers an advantage over the FFI as subjective responses to levels of exhaustion and physical activity are omitted. Additionally, the assessment of weight loss in the FFI may be difficult to interpret in patients who are sarcopenic but decompensated with large volume ascites. Despite these limitations, the FFI has been validated in cirrhotic populations and remains an acceptable alternative to the LFI in measuring physical frailty [39].

## 5. Conclusions

This is the first study to evaluate sarcopenia, physical frailty and REE together in patients with cirrhosis and portal hypertension. Sarcopenia and physical frailty present with similar patient characteristics but are associated with distinct differences in terms of BMI, REE and fuel utilisation. While both are associated with worse TFS when occurring individually, their detrimental impact is additive when they both occur in the same patient. The prognostic value of sarcopenia appears to be independent of physical frailty. Joint assessment of sarcopenia, frailty, and indirect calorimetry in patients with cirrhosis and portal hypertension can provide valuable information to improve prognostication and guide management beyond that obtained with one assessment alone [40].

## Figures and Tables

**Table 1:** Baseline clinical characteristics.

	*n* = 97
Median age (years)	57 (52–63)
Male sex (%)	69 (71)
Median BMI (kg/m^2^)	27 (24–32)
HCC (%)	19 (20)
Ascites (%)	65 (67)
Encephalopathy (%)	33 (34)
Child–Pugh class (%)	
A	20 (20)
B	40 (41)
C	34 (35)
Unknown	3 (3)
Median Child–Pugh score	9 (7–10)
Median MELD	16 (11–20)
Sarcopenia ^†^ (%)	23 (26)
Median TPMT/height (mm/m) ^†^	20 (17–22)
Frail ^‡^ (%)	38 (40)
Median FFI ^‡^	2 (1–3)
Median predicted REE ^§^ (kcal/day)	1637 (1440–1799)
Median measured REE ^§^ (kcal/day)	1695 (1459–1955)
Energy metabolism classification ^§^ (%)	
Hypermetabolic	13 (21)
Normometabolic	48 (76)
Hypometabolic	2 (3)
Median RQ ^§^ (IQR)	0.8 (0.7–0.9)
Median percentage fuel utilisation ^§^	
Carbohydrate	23 (8–40)
Fat	55 (39–69)
Protein	20 (17–23)
Subjective Global Assessment ^¶^ (%)	
A	17 (23)
B	41 (55)
C	17 (23)
Median MAC ^#^ (mm)	285 (266–330)
Median MAMC ^#^ (mm)	249 (224–271)
Median TSF ^#^ (mm)	12 (8–19)
Median hand grip strength ^^^ (kg)	28 (21–35)

Data are displayed as *n* (%) or median (IQR) as appropriate. **Abbreviations**: AIH, autoimmune hepatitis; BMI, body mass index; FFI, Fried Frailty Index; HCC, hepatocellular carcinoma; IQR, interquartile range; MAC, mid-arm circumference; MAFLD, metabolic dysfunction-associated fatty liver disease; MAMC, mid-arm muscle circumference; MELD, model for end-stage liver disease; PBC, primary biliary cholangitis; PSC, primary sclerosing cholangitis; REE, resting energy expenditure; RQ, respiratory quotient; TPMT, transversal psoas muscle thickness; TSF, triceps skinfold thickness. ^†^ Defined as TPMT < 16.8 mm/m. Not available in 7/97 patients. ^‡^ Defined as FFI ≥ 3. Not available in 1/97 patients. ^§^ Not available in 34/97 patients. ^¶^ Not available in 22/97 patients. ^#^ Not available in 24/97 patients. ^ Not available in 16/97 patients.

**Table 2:** Sarcopenia (TPMT/height) correlation analysis.

Category	Variable	Correlation Coefficient	*p*-Value
Demographics	Age	0.042	0.697
	BMI	0.275	**0.008**
Liver function	INR	−0.121	0.254
	Sodium	0.332	**0.001**
	Child–Pugh score	−0.214	**0.042**
	MELD score	−0.249	**0.017**
Frailty	FFI	−0.266	**0.016**
Anthropometry	MAC	0.416	**<0.001**
	MAMC	0.343	**0.003**
	Grip strength	0.285	**0.011**
Nutrition and energy expenditure	Measured REE	0.224	0.088
	Protein fuel utilisation	−0.310	**0.017**
	SGA	−0.363	**0.002**

Bold highlights which variables were statistically significant. **Abbreviations**: BMI, body mass index; FFI, Fried Frailty Index; INR, international normalised ratio; MAC, mid-arm circumference; MAMC, mid-arm muscle circumference; MELD, model for end-stage liver disease; REE, resting energy expenditure; SGA, Subjective Global Assessment; TPMT, transversal psoas muscle thickness.

**Table 3:** Frailty (FFI) correlation analysis.

Category	Variable	Correlation Coefficient	*p*-Value
Demographics	Age	0.138	0.205
	BMI	−0.028	0.798
Liver function	INR	0.254	**0.020**
	Sodium	−0.246	**0.024**
	Child–Pugh score	0.246	**0.024**
	MELD score	0.244	**0.025**
Sarcopenia	TPMT/height	−0.266	**0.016**
Anthropometry	MAC	−0.306	**0.007**
	MAMC	−0.312	**0.006**
	Grip strength	−0.424	**<0.001**
Nutrition and energy expenditure	Measured REE	−0.308	**0.014**
	Protein fuel utilisation	0.333	**0.008**
	SGA	0.241	**0.040**

Bold highlights which variables were statistically significant. **Abbreviations**: BMI, body mass index; FFI, Fried Frailty Index; INR, international normalised ratio; MAC, mid-arm circumference; MAMC, mid-arm muscle circumference; MELD, model for end-stage liver disease; REE, resting energy expenditure; SGA, Subjective Global Assessment; TPMT, transversal psoas muscle thickness.

**Table 4:** NFS and FNS patient characteristics.

	NFS *n* = 8	FNS *n* = 21	*p*-Value
Age (years)	54 (51–62)	57 (52–62)	0.448
BMI (kg/m^2^)	23 (21–27)	28 (24–33)	**0.032**
Child–Pugh score	9 (8–9)	9 (7–10)	0.867
MELD score	17 (13–26)	16 (12–17)	0.234
MAC (mm)	255 (235–275)	280 (252–325)	0.088
MAMC (mm)	229 (210–247)	238 (216–277)	0.349
Grip strength (kg)	28 (18–41)	27 (20–30)	0.435
REE (kcal/day)	1687 (1384–1785)	1561 (1267–1729)	0.396
Female sex (%)	5 (63)	15 (71)	0.675
Hypermetabolic ^†^ (%)	3 (60)	0 (0)	**0.017**
Ascites (%)	7 (88)	16 (76)	0.502
Encephalopathy (%)	2 (25)	10 (48)	0.269
Aetiology of cirrhosis (%)			0.450
Hepatitis C	3 (37)	10 (48)	
Hepatitis B	1 (13)	1 (5)	
Alcohol	2 (25)	4 (19)	
MAFLD	0 (0)	4 (19)	
AIH	0 (0)	1 (5)	
PBC	1 (13)	1 (5)	
Other	1 (13)	0 (0)	

Data are displayed as *n* (%) or median (IQR) as appropriate. Bold highlights which variables were statistically significant. **Abbreviations**: AIH, autoimmune hepatitis; BMI, body mass index; FNS, frail but not sarcopenic; MAC, mid-arm circumference; MAFLD, metabolic-associated fatty liver disease; MAMC, mid-arm muscle circumference; MELD, model for end-stage liver disease; NFS, not frail but sarcopenic; PBC, primary biliary cholangitis; REE, resting energy expenditure. ^†^ Indirect calorimetry available in 5/8 NFS and 11/21 FNS patients.

**Table 5:** Factors predictive of TFS on regression analysis.

	Univariate Analysis			Multivariate Analysis		
	HR	95% CI	*p*-Value	aHR	95% CI	*p*-Value
**Age (per year increase)**	1.002	0.979–1.024	0.893			
**Male sex (vs. female)**	1.039	0.638–1.691	0.877			
**BMI (per kg/m^2^ increase)**	1.003	0.960–1.047	0.908			
**Aetiology of liver disease**						
** Hepatitis C**	Ref					
** Hepatitis B**	2.150	0.820–5.635	0.120			
** Alcohol**	1.840	1.014–3.339	**0.045**	1.846	0.937–3.638	0.077
** MAFLD**	3.249	1.480–7.131	**0.003**	2.257	0.897–5.675	0.084
** AIH**	0.891	0.342–2.324	0.814			
** PSC**	1.700	0.649–4.456	0.280			
** PBC**	1.754	0.229–4.094	0.190			
**Albumin (per g/L increase)**	0.989	0.954–1.026	0.568			
**Ascites (vs. no ascites)**	2.374	1.405–4.012	**0.001**	0.764	0.374–1.560	0.459
**Hepatic encephalopathy (vs. no encephalopathy)**	1.772	1.113–2.821	**0.016**	2.051	1.211–3.474	**0.008**
**MELD (per point increase)**	1.114	1.080–1.148	**<0.001**	1.114	1.073–1.156	**<0.001**
**FFI (per point increase)**	1.240	1.046–1.469	**0.013**	0.945	0.756–1.182	0.623
**TPMT/height (per mm/m increase)**	0.901	0.845–0.962	**0.002**	0.930	0.868–0.996	**0.037**
**REE (per kcal/day increase)**	1.000	1.000–1.001	0.360			

Bold highlights which variables were statistically significant. **Abbreviations**: AIH, autoimmune hepatitis; BMI, body mass index; FFI, Fried frailty index; MAFLD, metabolic-dysfunction associated liver disease; MELD, model for end-stage liver disease; PBC, primary biliary cholangitis, PSC, primary sclerosing cholangitis; REE, resting energy expenditure; TPMT, transversal psoas muscle thickness.

## Data Availability

The data presented in this study are available on request from the corresponding author due to privacy and ethical restrictions.

## Supplementary Materials

The following supporting information can be downloaded at https://www.mdpi.com/article/10.3390/nu17243844/s1, Table S1: Reasons for incomplete assessment; Table S2: Sarcopenia measurements predictive of TFS on regression analysis; Table S3: Factors predictive of TFS on regression analysis using SMI as the measure of sarcopenia, Figure S1: Kaplan-Meier 6-month sensitivity analysis stratified by (a) sarcopenia status (b) frailty status and (c) frailty and/or sarcopenia status; Figure S2: Kaplan-Meier sensitivity analysis excluding HCC patients stratified by (a) sarcopenia status (b) frailty status and (c) frailty and/or sarcopenia status.

## Abbreviations

The following abbreviations are used in this manuscript:

REE	Resting energy expenditure
TPMT	Transversal psoas muscle thickness
FFI	Fried Frailty Index
TFS	Transplant-free survival
BMI	Body mass index
LT	Liver transplant
MELD	Model for end-stage liver disease
ACLF	Acute-on-chronic liver failure
HCC	Hepatocellular carcinoma
CT	Computed tomography
LFI	Liver Frailty Index
NFNS	Not frail and not sarcopenic
NFS	Not frail but sarcopenic
FNS	Frail but not sarcopenic
FS	Frail and sarcopenic
MAC	Mid-arm circumference
MAMC	Mid-arm muscle circumference
TSF	Triceps skinfold thickness
HGS	Hand grip strength
SGA	Subjective Global Assessment
EE	Energy expenditure
SD	Standard deviation
IQR	Interquartile range
VIF	Variance inflation factor
CI	Confidence interval
RQ	Respiratory quotient
INR	International normalised ratio
aHR	Adjusted hazard ratio
SMI	Skeletal muscle index
TPA	Total psoas muscle area
