# Supplementary material for: Interactions Between Sarcopenia, Physical Frailty and Resting Energy Expenditure in Cirrhosis and Portal Hypertension
Source: Nutrients. 2025 Dec 9;17(24):3844. doi: 10.3390/nu17243844 (PMC12735680; doi:10.3390/nu17243844)
Supplement: Supplementary file 1 [file nutrients-17-03844-s001.zip › nutrients-4014555-supplementary.pdf]

Supplementary Table S1. Reasons for incomplete assessment

**Sarcopenia CT measurements**	**n (%)**
Scan images unavailable	2 (29)
No scan performed	3 (43)
Umbilicus not included in scan	2 (29)
**Frailty assessment**	
Patient unwell	1 (100)
**Indirect calorimetry**	
Machine malfunction	3 (9)
Unable to scheduled prior to LT	15 (44)
Died	6 (18)
Patient factors (declined assessment, unwell or did not respond to dietitian)	8 (23)
Lost to follow up	2 (6)

Supplementary Table S2. Sarcopenia measurements predictive of TFS on regression analysis

	**Univariate analysis**		
	HR	95% CI	*p-value*
**TPMT/height (per mm/m increase)**	0.901	0.845-0.962	***0.002***
**TPA (per cm^2/m^2 increase)**	0.998	0.995-1.000	*0.054*
**SMI (per cm^2/m^2 increase)**	0.979	0.952-1.007	*0.146*

Bold highlights which variables were statistically significant. Abbreviations: SMI, skeletal muscle index; TPA, total psoas area; TPMT, transversal psoas muscle thickness

Supplementary Table S3. Factors predictive of TFS on regression analysis using SMI as the measure of sarcopenia

	**Univariate analysis**			**Multivariate analysis**		
	HR	95% CI	*p-value*	aHR	95% CI	*p-value*
**Age (per year increase)**	1.002	0.979-1.024	*0.893*			
**Male sex (vs. female)**	1.039	0.638-1.691	*0.877*			
**BMI (per kg/m^2 increase)**	1.003	0.960-1.047	*0.908*			
**Aetiology of liver disease**						
**Hepatitis C**	Ref					
**Hepatitis B**	2.150	0.820-5.635	*0.120*			
**Alcohol**	1.840	1.014-3.339	***0.045***	1.922	0.731-5.054	*0.185*
**MAFLD**	3.249	1.480-7.131	***0.003***	1.770	0.596-5.262	*0.304*
**AIH**	0.891	0.342-2.324	*0.814*			
**PSC**	1.700	0.649-4.456	*0.280*			
**PBC**	1.754	0.229-4.094	*0.190*			
**Albumin (per g/L increase)**	0.989	0.954-1.026	*0.568*			
**Ascites (vs. no ascites)**	2.374	1.405-4.012	***0.001***	0.812	0.382-1.723	*0.587*
**Hepatic encephalopathy (vs. no encephalopathy)**	1.772	1.113-2.821	***0.016***	2.260	1.308-3.905	***0.003***
**MELD (per point increase)**	1.114	1.080-1.148	***<0.001***	1.119	1.079-1.161	***<0.001***
**FFI (per point increase)**	1.240	1.046-1.469	***0.013***	0.955	0.752-1.213	*0.706*
**SMI (per cm^2/m^2 increase)**	0.979	0.952-1.007	*0.146*	0.989	0.955-1.024	*0.543*
**REE (per kcal/day increase)**	1.000	1.000-1.001	*0.360*			

Bold highlights which variables were statistically significant. Abbreviations: AIH, autoimmune hepatitis; BMI, body mass index; FFI, Fried frailty index; MAFLD, metabolic-dysfunction associated liver disease; MELD, model for end-stage liver disease; PBC, primary biliary cholangitis, PSC, primary sclerosing cholangitis; REE, resting energy expenditure; SMI, skeletal muscle index.

Supplementary Figure S1. Kaplan-Meier 6-month sensitivity analysis stratified by (a) sarcopenia status (b) frailty status and (c) frailty and/or sarcopenia status.

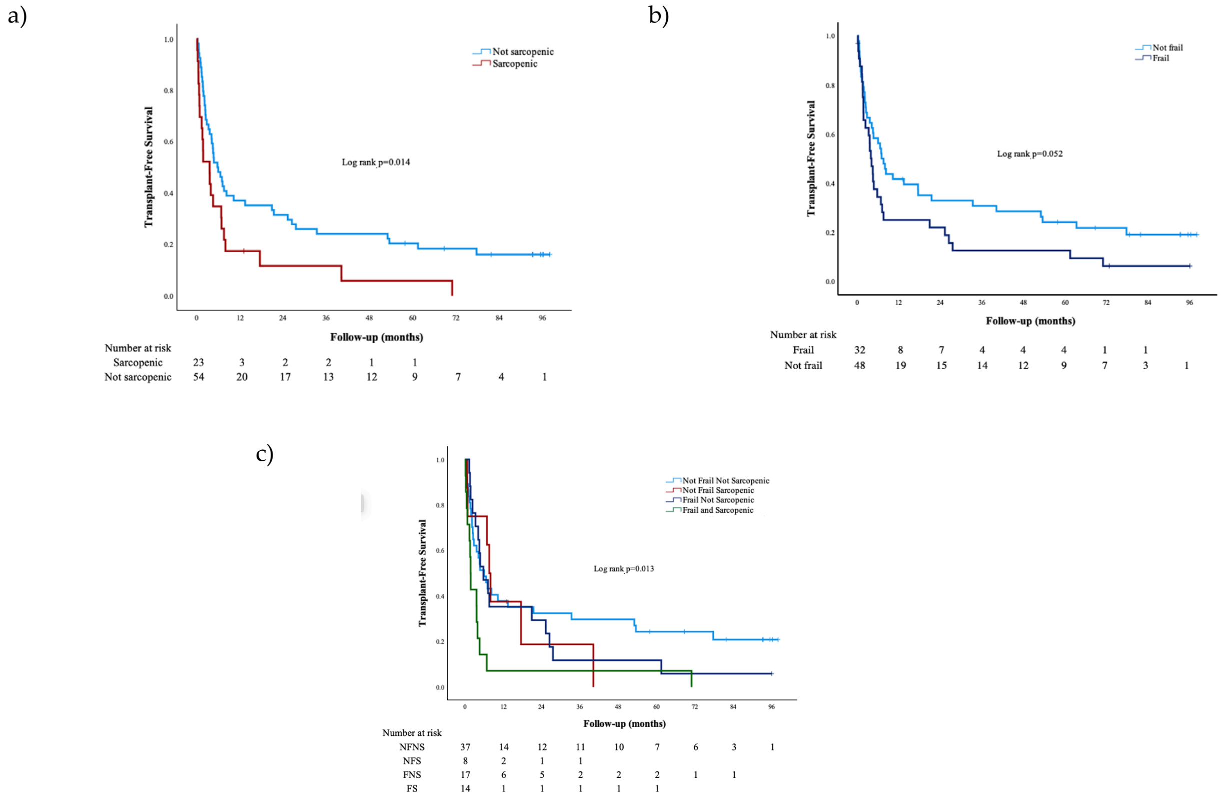

Abbreviations: NFNS, not frail and not sarcopenic; NFS, not frail but sarcopenic; FNS, frail but not sarcopenic; FS, frail and sarcopenic

Supplementary Figure S2. Kaplan-Meier sensitivity analysis excluding HCC patients stratified by (a) sarcopenia status (b) frailty status and (c) frailty and/or sarcopenia status.

a)

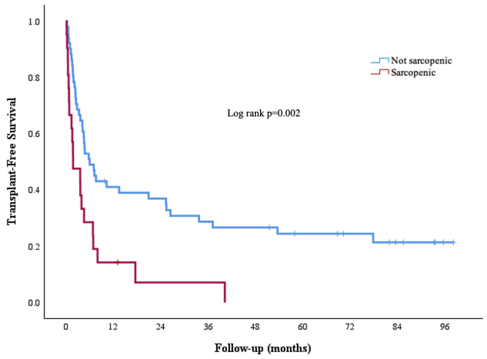

Number at risk									
Sarcopenic	51	20	18	14	13	10	8	5	1
Not sarcopenic	21	3	1	1					

b)

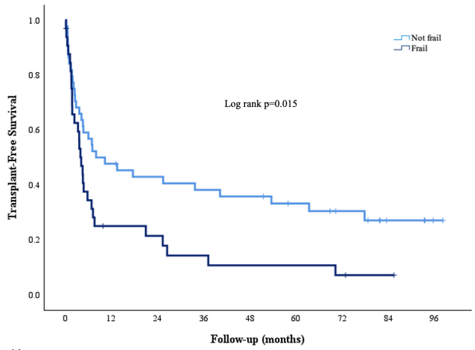

Number at risk									
Frail	44	21	18	16	14	12	9	4	1
Not frail	33	7	6	4	3	3	2	1	

c)

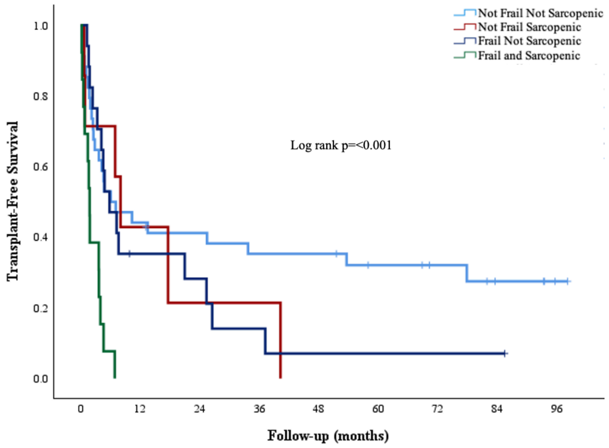

Number at risk									
NFNS	35	15	14	12	12	9	7	4	1
NFS	7	3	1	1					
FNS	17	5	4	2	1	1	1	1	
FS	13								

Abbreviations: NFNS, not frail and not sarcopenic; NFS, not frail but sarcopenic; FNS, frail but not sarcopenic; FS, frail and sarcopenic
